# Editorial: Transdiagnostic correlates of executive functions in psychiatric disorders

**DOI:** 10.3389/fnhum.2023.1268506

**Published:** 2023-08-21

**Authors:** Urs Heilbronner, Björn Albrecht

**Affiliations:** ^1^Institute of Psychiatric Phenomics and Genomics (IPPG), LMU University Hospital, LMU Munich, Munich, Germany; ^2^Clinical Child and Adolescent Psychology, Department of Psychology, Philipps-University Marburg, Marburg, Germany

**Keywords:** anorexia, bipolar disorder, depression, cognition, personality, children, adolescents, genetics

Executive Functions (EFs) are higher-order meta-cognitive capacities that control and coordinate mental processes implicated in many activities of daily life. EFs encompass domains such as the shifting of cognitive sets, inhibition, interference control and updating of working memory content, but also extend to broader personality constructs (Friedman and Robbins, 2021). Impaired EFs are implicated in many mental disorders, which is also reflected by research on an array of diagnoses presented in articles of this Research Topic. Our initial motivation for this Research Topic was that there is still a lack of evidence how EF deficits are shared across mental disorders or disorder subtypes, how these transdiagnostic impairments in EF may emerge and how they are mediated by underlying genetic, molecular, and neuronal mechanisms.

The five articles in this Research Topic highlight important areas of investigation for answering these questions. The importance of EFs in child and adolescent psychopathology is underscored by the four empirical research articles having a developmental focus.

Using behavioral genetics and latent variable analysis, Freis et al. investigate the relationships of psychopathology with both laboratory-based cognitive executive tasks and self-reported impulsivity. The authors conclude that both aspects of self-regulation have little phenotypic or genetic interrelation but are independently associated with transdiagnostic psychopathology. The intervention study by Prillinger et al. found in children with autism-spectrum disorder receiving intensive neurofeedback training of slow cortical potentials that post-treatment changes in contingent negative variation (CNV), a wellstudied EEG indicator of executive processes, was moderated by impulsivity symptoms. This indicates that the relationship of personality and cognitive laboratory measures of EFs may manifest itself at the neuronal level but is not detected otherwise. Using rating-scales to assess EFs, Albermann et al. show that there are only subtle deficits in EFs in depression, which are however associated with borderline personality features, pointing to another fascinating interaction of personality and cognition in psychopathology. In a study of both unipolar and bipolar depression, Peterson et al. examined associations of deficits in EFs with common and specific mood symptoms using structural equation modeling. Their analyses reveal both types of mood symptoms to be associated with EFs, pointing to the overarching importance of EFs in transdiagnostic psychopathology. Finally, Thomas et al. present a carefully conducted review of transdiagnostic EF deficits of behavioral and neuroimaging findings in anorexia nervosa and obsessive compulsive disorder, two forms of psychopathology characterized by extensive comorbidity.

Although the presented evidence is not conclusive, we hope that readers of this Research Topic will find the collection of articles inspiring for their own research and instructive for therapeutic practice. Special thanks go to all contributing authors, reviewers, and study participants.

## Author contributions

UH: Conceptualization, Writing—original draft, Writing—review and editing. BA: Conceptualization, Writing—original draft, Writing–review and editing.
